# *Streptococcus pneumoniae* serotype 3 genotypes in invasive isolates from Colombia

**DOI:** 10.7705/biomedica.5407

**Published:** 2021-06-15

**Authors:** Zonia Katerin Alarcón, Carolina Duarte, Olga Sanabria, Jaime Moreno

**Affiliations:** 1 Grupo de Microbiología, Instituto Nacional de Salud, Bogotá, D.C., Colombia Instituto Nacional de Salud BogotáD.C Colombia

**Keywords:** *Streptococcus* pneumoniae, electrophoresis, gel, pulsed-field, Colombia, Streptococcus pneumoniae, Colombia, electroforesis en gel de campo pulsado, Colombia

## Abstract

**Introduction:**

*Streptococcus pneumoniae* serotype 3 is an important cause of pneumonia, bacteremia, and meningitis.

**Objective:**

To establish the circulating genotypes of *S. pneumoniae* serotype 3 isolates recovered from the invasive disease between 1994 to 2015 in Colombia.

**Materials and methods:**

Of the 365 *S. pneumoniae* serotype 3 isolates recovered through the laboratory national surveillance program, 117 isolates were analyzed. Pulsed-field gel electrophoresis was used for genotyping, and multilocus sequence typing was determined in representative isolates.

**Results:**

The frequency of this serotype increased from 2.7% between 1994 and 1998 to 9.1% between 2011 and 2015 (p=0.000); 91.7% of the isolates showed a genetic similarity greater than 77% and were related to the Netherlands^3^-31(PMEN31) clone CC180. Several subtypes were identified, two of which showed antimicrobial resistance.

**Conclusion:**

In Colombia, the pneumococcal population of the capsular type 3 shows a continuous and homogeneous circulation relating to the clonal group ST-180.

## Resumen

**Introducción.:**

El serotipo 3 de *Streptococcus pneumoniae* es una causa importante de neumonía, bacteriemia y meningitis.

**Objetivo.:**

Establecer los genotipos circulantes de aislamientos del serotipo 3 de *S. pneumoniae* recuperados de muestras de enfermedad invasiva de 1994 a 2015 en Colombia.

**Materiales y métodos.:**

Se analizaron 117 de los 365 aislamientos del serotipo 3 de *S. pneumoniae* recuperados del programa nacional de vigilancia por el laboratorio. El genotipo se estableció con electroforesis en gel de campo pulsado y la tipificación se llevó a cabo mediante secuenciación multilocus en aislamientos representativos.

**Resultados.:**

La frecuencia de este serotipo aumentó de 2,7 % entre 1994 y 1998 a 9,1 % entre 2011 y 2015 (p=0,000). El 91,7 % de los aislamientos evidenció una similitud genética superior al 77 % y se relacionó con el clon CC180 de Netherlands3-31 (PMEN31). Se identificaron varios subtipos, dos de los cuales mostraron resistencia a los antimicrobianos.

**Conclusión.:**

En Colombia, la población neumocócica del tipo capsular 3 tiene una circulación continua y homogénea relacionada con el grupo clonal ST-180.

*Streptococcus pneumoniae* causes serious invasive diseases such as pneumonia and meningitis in young children worldwide ^1^. In 2015, the worldwide mortality rate due to *S. pneumoniae* was 45 deaths per 100,000 children from one to 59 months. A global study of the burden of *S. pneumoniae* disease in children estimated 294,000 cases for the Americas during the period 2010 to 2015, of which 5,700 died, however, an average annual reduction of 8% in deaths was observed related to the use of pneumococcal conjugate vaccines ^2^.

Pneumococcal capsule allows the bacterium to be classified in more than 100 serotypes according to its antigenic composition ^3^. *Streptococcus pneumoniae* serotype 3 is characterized by its high virulence, great invasive capacity, high mortality, and susceptibility to clinical antibiotics ^4^. Molecular typing studies of *S. pneumoniae* serotype 3 have shown low genetic diversity among the isolates, which is associated with the short duration of nasopharyngeal carriage and its propensity to cause an invasive infection ^5^.Molecular characterization has enabled the identification of the Netherlands^3^-31(PMEN31) clone CC180 as the predominant lineage for this serotype ^6^^,^^7^ demonstrating its distribution and clonality in different parts of the world such as Japan ^8^, Denmark ^9^, North America ^10^, South America ^11^, and South Africa ^12^.

Since 1994, Colombia has been monitoring the distribution of serotypes and antimicrobial susceptibility of invasive diseases isolates recovered from children under five years old. This program was extended in 1996 to the general population, thus forming a single national surveillance program that is part of the Latin America Network Surveillance System for the Bacterial Agents Responsible for Pneumonia and Meningitis (SIREVA II) project ^13^^,^^14^. Based on reports from 2006 to 2018, *S. pneumoniae* type 3 in Colombia represents 6.37% of isolates recovered in children under 5 years old and 10.77% in adults ^15^. Published data describing the distribution of serotypes during 2000 and 2010 consigned that serotype 3 was associated with 5.5% (98/1,775) invasive isolates and presented an increasing frequency of 4.4% in the years 2007 to 2008 to 7.2% from 2009 to 2010 (p=0.01) ^16^. Colombia adopted the PCV10 universal vaccination in 2011 in a 2 + 1 scheme at 2, 4, and 12 months for children less than one-year-old through the public health system ^17^.

The aim of this study was to establish the genetic relationships of *S. pneumoniae* serotype 3 isolates causing invasive diseases recovered from 1994 to 2015 to provide information and knowledge about the structure of the population.

## Materials and methods

From 1994 to 2015, we recovered 365 *S. pneumoniae* serotype 3 invasive isolates from laboratory surveillance. The isolates were typified using the Quellung reaction and the antimicrobial susceptibility to penicillin, ceftriaxone, chloramphenicol, trimethoprim-sulfamethoxazole, vancomycin, erythromycin, and tetracycline was determined by both the disk diffusion method (Kirby- Bauer) and broth microdilution to determine minimal inhibitory concentrations (MIC) according to the criteria of the Clinical and Laboratory Standards Institute (CLSI) guidelines for each year ^18^. Isolates were sent mainly from Bogotá (44.4%), Antioquia (21.4%), and Valle del Cauca (9.3%), and the remaining 21.1% from other 20 political divisions.

We calculated a representative sample of the 365 isolates with the formula for the point estimate of prevalence assuming a prevalence of 50%, a maximum expected difference of 15% (± 7.5%), and an alpha error of 5%. The representative sample was 117 isolates, of which 95 were randomly selected proportionally to the frequencies observed in each year and 22 that presented resistance to at least one antibiotic.

Genotyping was carried out by pulsed-field gel electrophoresis (PFGE) using the *Sma*I restriction enzyme. The R6 strain and 12 international clones of the Pneumococcal Molecular Epidemiology Network (PMEN) including the Netherlands3-31(PMEN31) clone CC180 were used as controls ^6^^,^^19^. The electrophoretic patterns were analyzed using the GelCompar program for generating dendrograms and ≥75% genetic similarity was assumed for the definition of clonality ^19^. Five isolates were selected according to the analysis of the dendrogram generated by PFGE for molecular typing by multilocus sequence typing (MLST) performed following the protocol of Enright, *et al*. ^20^. The sequence type of each isolate was determined using the software available on the MLST website (https://pubmlst.org/spneumoniae/).

### Statistical analysis

We made a descriptive analysis. Variables were described using relative or absolute frequencies of the demographic and clinical variables of the *S. pneumoniae* serotype 3 isolates. For the frequency of serotype 3, the data were grouped into four periods, two of five years: 1994-1998, 1999-2003, and two of six years: 2004-2009, 2010-2015. The relationships or associations between the variables were evaluated using the X^2^ or Fisher’s test. An association was considered significant if it had a value of p≤0.05. The Epi-Info 7 statistical program was used.

## Results

The 365 isolates of serotype 3 represented 6.1% of the total isolates received during the period under study; their distribution over time periods is shown in table 1. We observed a significant difference in the frequency of serotype 3 isolates, which increased from 2.7% (20/741) in 1994-1998 to 9.1% (223/2,448) in 2010-2015 (p=0.000). In children under five years the frequency was 3.3% (30/902) while in patients over 50 it was 11.9% (173/1,449). The main diagnosis of patients with serotype 3 was pneumonia (n=132, 36.2%) followed by sepsis (n=100, 27.4%). From all the pneumonia cases, serotype 3 was identified in 6.5% (132/2030) of them, and in sepsis cases, it was recovered in 9.5% (100/1,404) (table 1).

**Table 1:** Distribution of and *Streptococcus pneumoniae* serotype 3 isolates recovered from surveillance in Colombia, 1994-2015

*Streptococcus pneumoniae*		Period (years)				Age groups (years)					Diagnosis					
		1994- 1998	1999- 2003	2004- 2009	2010- 2015	<2	2-5	6-19	20-49	>50	ND	MBA	Pneumonia	Sepsis	Other	ND
		n	n	n	n	n	n	n	n	n	n	n	n	n	n	n
		(%)	(%)	(%)	(%)	(%)	(%)	(%)	(%)	(%)	(%)	(%)	(%)	(%)	(%)	(%)
Serotype 3, (n)	365	20	36	86	223	56	30	33	53	173	20	69	132	100	35	29
		(5.5)	(9.9)	(23.6)	(61.1)	(15.3)	(8.2)	(9.0)	(14.5)	(47.4)	(5.5)	(18.9)	(36.2)	(27.4)	(9.6)	(7.9)
			1,010	1,786	2,448	1,638	902	751	1,051	1,449	194	1,758	2,030	1,404	424	369
All serotypes, (n)	5,985	741	(12.4) (16.9)	(29.8)	(40.9)	(27.4)	(15.1)	(12.5)	(17.6)	(24.2)	(3.2)	(29.4)	(33.9)	(23.5)	(7.1)	(6.2)
Serotype 3/all	6.1	2.7	3.6	4.8	9.1	3.4	3.3	4.4	5.0	11.9	10.3	3.9	6.5	7.1	8.3	7.9
serotypes (%)																

MBA: Bacterial meningitis, ND: No data

As regards the seven antibiotics evaluated, the 365 isolates were susceptible to penicillin, ceftriaxone, and vancomycin. Twenty-two isolates were resistant to at least one antibiotic (chloramphenicol, trimethoprim- sulfamethoxazole, erythromycin, and tetracycline), four of which had reduced susceptibility to penicillin (MIC=0.125 µg/ml) and six were multi-resistant (to chloramphenicol, erythromycin, and tetracycline) (table 2).

**Table 2:** Distribution of the restriction patterns of *Streptococcus pneumoniae* serotype 3 isolates received in the surveillance program in Colombia, 1994-2015

Pattern PFGE		n	Period (years)				Age groups			Diagnosis						Antimicrobial sensitivity									ST
			1994- 1998	1999- 2003	2004- 2009	2010- 2015	<2	3- 5	6- 19	20- 49	>50	ND	MBA	Pneumonia	Sepsis	Others	ND	Susceptible	RSP	Resistance				Multi resistance	
																				Chlor	STX	Ery	Te		
A		107	6	12	23	66	22	14	7	15	43	6	23	40	25	12	7	89	2	7	2	7	13	5	
Sub-	A1	35	1	2	6	26	4	6	1	8	13	3	9	12	10	1	3	34	1	0	0	0	0	0	180
Patterns	A2	26	2	3	11	10	5	4	2	2	12	1	5	10	6	5		25	1	0	0	0	0	0	180
A	A3	8		3	1	4	3		1		3	1		4	3	1		8	0	0	0	0	0	0	-
	A4	8				8	1				6	1	1	4	2		1	0	0	7	0	6	8	5	180
	A5	7		2	1	4	2		2	2	1		1	3	1	2		0	0	0	2	1	4	0	180
	A6	5				5		2		1	2			3		1	1	5	0	0	0	0	0	0	9162
	A7	4			1	3	2			1	1		2			1	1	4	0	0	0	0	0	0	-
	A8	4	1	1		2					4			1	1	1	1	3	0	0	0	0	1	0	-
	A9	3	1		1	1	1			1	1		1	1	1			3	0	0	0	0	0	0	-
	A10	3	1		2		2	1					2	1				3	0	0	0	0	0	0	-
	A11	2		1		1	1		1				2		1			2	0	0	0	0	0	0	-
	A12	1				1		1						1				1	0	0	0	0	0	0	-
	A13	1				1	1			1	1							1	0	0	0	0	0	0	-
B		3	1	1		1	1		1		2			1				0	1	0	1	0	3	1	-
C		2				2			2				2	1			1	1	1	0	0	0	0	0	-
D		2				2	1			1		1			2			2	0	0	0	0	0	0	-
NR		3	1		1	1	1						1	2				3	0	0	0	0	0	0	-
Total n		117	8	13	24	72	25	14	8	17	46	7	26	44	27	12	8	95	4	7	3	7	16	6	ST: 180 9162

MBA: Bacterial meningitis; ND: No data; NR: Not related; RSP: reduced susceptibility to penicillin; Chlor: Chloramphenicol; STX: Trimetroprim-sulfamethoxazole; Ery: Erythromycin; Te: Tetracycline, ST: Sequence type

From the 117 isolates characterized by PFGE, 107 (91.4%) were grouped in electrophoretic pattern A with 13 subtypes (A1-A13), they had genetic similarity greater than 77%, and were related to the Netherlands^3^-31-ST-180 clone (table 2 and supplementary figure). Subtype A1 grouped 35/107 (32.7%) isolates with a 98% similarity of which 26/35 (74.2%) were recovered between 2010 and 2015 mainly from patients with pneumoniae (n=12, 34.2%) and adults over 50 years (n=13, 37.1%). Only one isolate showed reduced susceptibility to penicillin.

As regards subtype A2, 26/107 (24.3%) isolates had a 96.7% genetic similarity and were recovered mainly in the last two periods under study: 2004-2015 (n=21, 80.7%) from patients over 50 years old (n=12, 46.2%) with pneumonia diagnosis (n=10, 38.4%) and one isolate showed reduced susceptibility to penicillin.

Subtype A3 with 8/107 (7.4%) isolates had a 96.8% genetic similarity. All the isolates were susceptible to the antibiotics evaluated. 

Subtype A4 was found in 8/107 (7.4%) isolates with a 100% similarity, mostly recovered from 2010 to 2015 of adults over 50 years (n=6, 75%) and patients with pneumonia (n=4 50%). Three isolates had resistance to two antibiotics (two to chloramphenicol and tetracycline and one to erythromycin and tetracycline) while five isolates (62.5%) were multiresistant to chloramphenicol, erythromycin, and tetracycline. The isolates were recovered in different political divisions. 

Subtype A5 was identified in 7/107 (6.5%) isolates with a 93% genetic similarity; all of them were resistant to at least one of the antibiotics evaluated. 

Subtype A6 grouped 5/107 (4.6%) isolates with a 91% similarity. Subtypes A7-A13 were identified in 18/107 (16.8%) isolates, all of which were susceptible to the antibiotics. One isolate of subtypes A1, A2, A4, A5, and A6 was selected by MLST. ST180 was identified in subtypes A1, A2, A4, and A5. In A6, the isolate was related to ST-9162, a single locus variant in the *aroE* allele of ST-180 (supplementary figure). 

## Discussion

In Colombia, serotype 3 represents 6.1% of isolates recovered from invasive diseases, most are susceptible to antibiotics for clinical use and were isolated mainly from patients over 50 years. The molecular typing of the isolates characterized in this study showed genetic homogeneity related to the Netherlands3-31/ST180 clone.

Data analyses of SIREVA II reports from 2000 to 2014 ^21^ was performed to establish the frequencies of serotype 3 in the region. According to the analysis, Brazil (7.35%), Chile (5.42%), and México (5.23%) presented similar frequencies as those found in Colombia. Higher serotype 3 frequencies were observed in Uruguay (10.81%) and Costa Rica (10.32%) while lower frequencies were detected in Guatemala (1.42%) and Honduras (0.74%). A recent meta-analysis to establish epidemiological data in pediatric pneumococcal pneumonia in Latin America from 2000 to 2016 showed low frequencies in Paraguay (0.8%) and Chile (0.9%) and high frequencies in Uruguay (6.5%) and Venezuela (8.1%) ^22^.

In the United States, the frequency of pneumococcal disease associated with serotype 3 before the introduction of PCV13 was 4% in children and 9% in adults ^23^. In European countries such as Italy, serotype 3 was the most frequent in patients above the age of 45 years accounting for 17.3% of the isolates ^24^. In Spain, from 2010 to 2013, serotype 3 was one of the most frequent invasive disease serotypes with 12% of isolates recovered from children under five years old and 18% from children over five years old ^25^ while in Japan, it was the main serotype with a frequency of 14.7% in isolates recovered from adults between 2006 and 2014 ^26^.

In this study, *S. pneumoniae* serotype 3 isolates were recovered mainly from patients older than 50 years old (table 1), which is similar to findings from other countries that reported the highest frequency in older adults. Similarly, serotype 3 was recovered most frequently in patients diagnosed with pneumonia (table 1) in agreement with findings from other studies ^27^^,^^28^. Capsular type 3 has been associated with complicated pneumonia, empyema, and necrotizing pneumonia with high mortality rates in children over five years old and adults ^27^^,^^29^^,^^30^. The second most frequent diagnosis associated with the isolates was sepsis. Sepsis occurs in approximately 30% of cases of community-acquired pneumonia ^31^ and serotype 3 has been independently associated with septic shock in patients with pneumococcal pneumonia ^32^. This finding could be related to the ability of capsular type 3 to produce high concentrations of capsular polysaccharides to overcome host immunity and proliferate more efficiently than other pneumococcal serotypes ^28^.

*Streptococcus pneumoniae* serotype 3 is characterized as susceptible to most antimicrobials for clinical use ^4^. In this study, four (1.1%) isolates were reduced susceptibility to penicillin (MIC=0.125 - 0.250 μg/ml). Penicillin resistance is related to modifications in the amino acid motifs conserved in penicillin-binding proteins encoded by the *pbp*1a, *pbp*2b, and *pbp*2x genes ^33^. Alterations in the *pbp*2x gene have been identified in serotype 3 isolates with intermediate penicillin resistance ^34^. Additionally, a serotype 3 penicillin-resistant isolate (MIC=1 μg/ml) was generated by genetic recombination of the capsule locus 3 of a susceptible isolate with the Taiwan23F-15 ST-242 clone ^35^.

We found six multiresistant isolates to tetracycline, chloramphenicol, and erythromycin*.* Resistance to these antibiotics has mainly been associated with the *tetM, cat,* and *ermB/Mefe* genes, respectively ^7^^,^^8^^,^^36^, usually located on mobile genetic elements such as Tn916 and Tn5253 ^37^. Future studies are necessary to determine if these mobile elements are present in these isolates.

In Colombia, genetic homogeneity was observed in the serotype 3 isolates related to the Netherlands^3^-31-ST-180 clone. ST-180 is the most frequent typing for serotype 3 and is characterized by its susceptibility to antibiotics for clinical use and its wide geographical distribution ^8^^,^^10^^,^^11^^,^^20^. Although ST-180 is still the most common, other clonal groups such as ST-458 have also been reported for this capsular type, which is the predominant lineage in the serotype 3 isolates recovered in South Africa ^12^, as well as ST-4909, which is the second most frequent sequence type in Mexican isolates ^38^, and ST-260, which was found in 41.9% of serotype 3 isolates in Spain ^39^. Additionally, as a result of possible capsular switching events, the presence of capsular type 3 has been reported in other clones of worldwide distribution including Spain^23F^-1-3, Taiwan^23F^-15-3, Poland^6B^-20-3, Portugal^19F^-21-3, Spain^23F^-1-3, Netherlands^14^-35-3, Netherlands^15^-37-3, and Netherlands^7F^-39-3 (https://pubmlst.org/spneumoniae/).

In spite of the clonality of the isolates of serotype 3, we observed variability in electrophoretic patterns that allowed subdivision into subtypes. The subtype A1 contained the isolates recovered in the last study period, which suggests that this subtype is the one currently circulating and should be evaluated if it presents genetic differences favoring its dispersion and successful behavior. Subtypes A4 and A5 contained antibiotic-resistant isolates and were recovered in recent years. This finding suggests possible genetic recombination events through the acquisition of mobile genetic elements conferring resistance mechanisms ^7^. These subtypes may represent emerging clonal lineages that could diminish the therapeutic options against invasive pneumococcal disease associated with this capsular type. Based on the whole-genome analysis, recently, Azarian, *et al.*, described 3 lineages for ST180: Clade Iα, clade Iβ, and clade II. Clade II is an emerging lineage that is increasing its population size and prevalence of antibiotic resistance by the acquisition of Tn916 ^40^. The subtypes A4 and A5 found in this study may be related to this lineage due to its phenotypic and genotypic characteristics.

The global pneumococcal sequencing project (GPS, http://www. pneumogen.net/gps/) analyzed 20,027 *S. pneumoniae* genomes and defined 621 global pneumococcal sequence clusters (GPSC) of which only the GPSC12 included susceptible and resistant isolates expressing serotype 3 and ST180 ^41^. Here we did not perform whole-genome sequencing to define the GPSC of isolates, however, we expect to perform this type of analysis in this pneumococcal population in the future.

Our study limitations include the passive surveillance of bacterial meningitis and pneumonia, as not all cases can be detected, but it is sustainable over time and it provides an overview of the general distribution of isolates, as well as important information for monitoring serotypes associated with invasive pneumococcal disease circulating in the country.

The present study described the genetic characteristics of the invasive serotype 3 isolates in the pneumococcal population in Colombia. It demonstrated their clonality and identified predominant subtypes in the most recent sample period, which may represent important lineages with antimicrobial-resistant characteristics. Therefore, it is necessary to carry out continuous monitoring and additional studies to determine the evolution and impact of *S. pneumoniae* serotype 3 isolates.

## Figures and Tables

**Figure:**
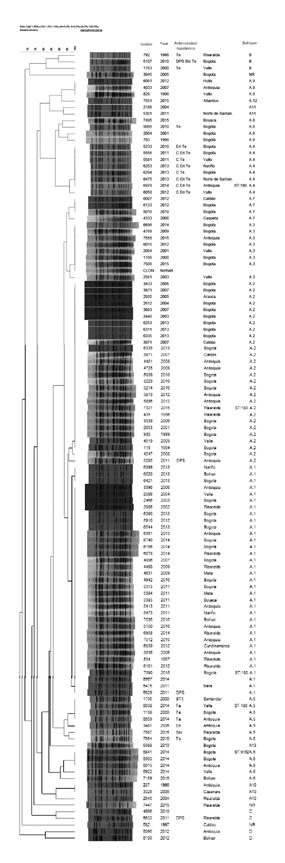
Dendrogram of isolates of S. pneumoniae serotype 3 received in the surveillance program in Colombia, 1994-2015
